# Adopting AMSTAR 2 critical appraisal tool for systematic reviews: speed of the tool uptake and barriers for its adoption

**DOI:** 10.1186/s12874-022-01592-y

**Published:** 2022-04-10

**Authors:** Ruzica Bojcic, Mate Todoric, Livia Puljak

**Affiliations:** 1 Institute of Emergency Medicine of Karlovac County, Karlovac, Croatia; 2 University Hospital Split, Split, Croatia; 3 Center for Evidence-Based Medicine and Health Care, Catholic University of Croatia, Ilica 242, 10000 Zagreb, Croatia

## Abstract

**Background:**

In 2007, AMSTAR (A MeaSurement Tool to Assess systematic Reviews), a critical appraisal tool for systematic reviews (SRs), was published, and it has since become one of the most widely used instruments for SR appraisal. In September 2017, AMSTAR 2 was published as an updated version of the tool. This mixed-methods study aimed to analyze the extent of the AMSTAR 2 uptake and explore potential barriers to its uptake.

**Methods:**

We analyzed the frequency of AMSTAR or AMSTAR 2 use in articles published in 2018, 2019 and 2020. We surveyed authors who have used AMSTAR but not AMSTAR 2 in the analyzed time frame to identify their reasons and barriers. The inclusion criterion for those authors was that the month of manuscript submission was after September 2017, i.e. after AMSTAR 2 was published.

**Results:**

We included 871 studies. The majority (*N* = 451; 52%) used AMSTAR 2, while 44% (*N* = 382) used AMSTAR, 4% (*N* = 31) used R-AMSTAR and others used a combination of tools. In 2018, 81% of the analyzed studies used AMSTAR, while 16% used AMSTAR 2. In 2019, 52% used AMSTAR, while 44% used AMSTAR 2. Among articles published in 2020, 28% used AMSTAR, while AMSTAR 2 was used by 69%.

An author survey indicated that the authors did not use AMSTAR 2 mostly because they were not aware of it, their protocol was already established, or data collection completed at the time when the new tool was published. Barriers towards AMSTAR 2 use were lack of quantitative assessment, insufficient awareness, length, difficulties with a specific item.

**Conclusion:**

In articles published in 2018-2020, that were submitted to a journal after AMSTAR 2 tool was published, almost half of the authors (44%) still used AMSTAR, the old version of the tool. However, the use of AMSTAR has been declining in each subsequent year. Our survey indicated that editors and peer-reviewers did not ask the authors to use the new version of the tool. Few barriers towards using AMSTAR 2 were identified, and thus it is anticipated that the use of the old version of AMSTAR will continue to decline.

**Supplementary Information:**

The online version contains supplementary material available at 10.1186/s12874-022-01592-y.

## Background

In 2007, AMSTAR (A MeaSurement Tool to Assess systematic Reviews), a critical appraisal tool for systematic reviews (SRs), was published, and it has since become one of the most widely used instruments for SR appraisal [1]. In September 2017, AMSTAR 2 was published as an updated version of the tool, which was also adopted to enable a more detailed appraisal of SRs that include randomized or non-randomized studies of interventions in health care, or both [2].

AMSTAR 2 has 16 items, compared to 11 items in the original AMSTAR. Furthermore, based on the information published in the research literature, AMSTAR 2 authors reported that AMSTAR 2 has simpler response categories than the original tool; it includes a more comprehensive user guide and has instructions for making an overall rating based on weaknesses identified in critical domains [1, 2].

Based on the information published in the research literature, the use of AMSTAR 2 for appraising SRs also requires more time than AMSTAR. Banzi et al. reported that for five raters with variable experience mean time to complete AMSTAR was 5.8 min [3], while Pieper et al. reported that four raters of variable experience had a mean time for completing AMSTAR 2 of 18 min [4]. These preliminary results indicate that using AMSTAR 2 is much more time-consuming, particularly if there are many SRs to rate in a research project. Despite the introduction of the new, updated tool, we have noticed that researchers still frequently use the first version of the AMSTAR. This study aimed to analyze the extent of the AMSTAR 2 uptake within the first 3 years after its publication and explore potential barriers to its uptake.

## Methods

### Study design

This was a mixed-methods study that consisted of two parts. The first part was a bibliographic analysis of studies that have used AMSTAR or AMSTAR 2 for appraisal of SRs and that were published between January 1, 2018, and December 31, 2020. The second part of the study was a survey of authors who have used the AMSTAR but not AMSTAR 2 in the analyzed time frame.

### Ethics

For the second part of the study, Ethics Committee of the Catholic University of Croatia approved the research protocol. All participants gave their written informed consent for participation via e-mail. All methods were carried out in accordance with relevant guidelines and regulations, including the Ethics Code of the Catholic University of Croatia and the Declaration of Helsinki.

### Study eligibility

We included original studies that have used AMSTAR or AMSTAR 2 for appraisal of included SRs. We excluded studies that have only mentioned AMSTAR or AMSTAR 2 but did not report that they used the tool for appraising included SRs, and studies that reported that their study was prepared in line with the tool. We also excluded studies that were devoted specifically to characterizing AMSTAR 2.

### Search and screening

For the first part of the study, we searched MEDLINE and Embase via OVID to find studies that have used the word AMSTAR. We used the following search strategy: (((AMSTAR) OR (AMSTAR-2)) OR (AMSTAR 2)) OR (R-AMSTAR). This broad strategy was used to retrieve studies mentioning any version of the name of the AMSTAR tool, anywhere in the text.

We exported retrieved bibliographic records into EndNote X5 (Clarivate Analytics, London, UK) reference management software and deleted duplicates. We screened bibliographic records to include studies that used AMSTAR or AMSTAR 2 to critically appraise SRs (for example, overviews of SRs, i.e., umbrella reviews, or methodological studies appraising the quality of SRs). Two authors screened titles and abstracts retrieved by database searching, retrieved potentially eligible studies in full text, and screened those manuscripts in full-text again independently.

### Data extraction

One author extracted data, and another author verified data extraction. For eligible studies, we extracted the following information: journal, a month of submission to a journal, month of manuscript acceptance, a month of online publication, use of AMSTAR or AMSTAR 2, type of publication (overview of SRs, methodological study). The source of information (reference) for using AMSTAR or AMSTAR 2 were also extracted.

### Survey

In May 2020 (for articles published in 2018-2019) and January/February 2022 (for articles published in 2020), we contacted via e-mail corresponding authors of eligible studies who did not use AMSTAR 2 and sent them a short survey. We used only e-mail addresses provided in published manuscripts; we did not make any attempt to find alternative e-mail addresses if an e-mail would return undelivered or if we did not receive a response. Each potential participant received two reminders 1 week apart.

Inclusion criteria for those authors were that the month of manuscript submission was after September 2017, i.e. after AMSTAR 2 was published. Text of the e-mail is available in Supplementary file 1.

To preserve participants’ anonymity, individuals were invited to answer the survey questions in a survey placed on Google Forms. In the invitation e-mail, participants were informed about the purpose of this study and asked whether they are aware of the AMSTAR 2, reasons why they did not use AMSTAR 2 instead of AMSTAR, whether editors or peer-reviewers suggested they should use AMSTAR 2, and asked if they can recognize any barriers for the uptake of the AMSTAR 2 that they have experienced, or that someone else might experience. Additionally, they were asked about years of experience with evidence synthesis or methodological research.

After sending the survey out, we were contacted by several authors from China, who indicated that they were not able to access the survey. Thus, we screened affiliations of all corresponding authors that were supposed to be contacted in the survey, and we sent the survey questions via e-mail to corresponding authors with affiliations in China.

### Data analysis

We conducted descriptive data analysis using frequencies and percentages; for data analysis, we used MedCalc (MedCalc Software bv., Ostend, Belgium).

## Results

### Bibliometric analysis

**Fig. 1:**
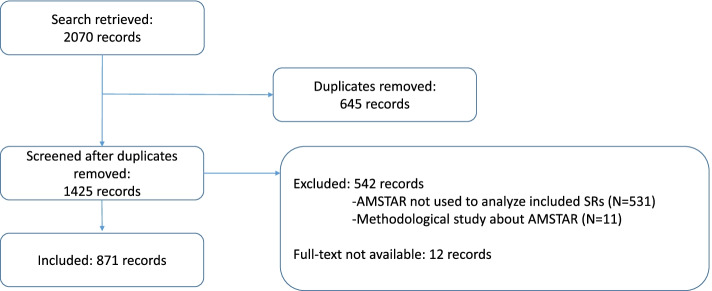
Flow chart of the studies

We retrieved 2070 records from databases. After removing duplicates, we screened 1425 records. We excluded 542 records that were not eligible; the excluded studies did not use the analyzed tool to assess SRs in original articles. The majority of excluded studies only mentioned that their review was prepared according to AMSTAR. We could not analyze 12 studies because the full text was not available. The list of excluded and unavailable studies, with reasons, is available in Supplementary file 2. We included 871 studies. Figure 1 shows study flow chart. All raw data collected within the study are reported in Supplementary file 3.

Most of the studies (70%) were overviews of systematic reviews (OSR), followed by methodological studies appraising SRs (18%) (Table 1). The majority of the reports were full-text manuscripts published in scholarly journals (Table 1). The studies were published in 513 different journals, most frequently in the *BMJ Open*, *Systematic Reviews*, *PLoS One*, *Medicine*, and *Journal of Clinical Epidemiology* (Table 1).

**Table 1:** Characteristics of included studies (*N* = 871)

Variable	N (%)^**a**^
Study design	
Overview of systematic reviews	612 (70)
Methodological study	160 (18)
Protocol for an overview of systematic reviews	61 (7.0)
Guideline	29 (3.3)
Policy brief with evidence synthesis	1 (0.01)
Health Technology Assessment	1 (0.01)
Protocol for a methodological study	1 (0.01)
Methodological study and overview of systematic reviews	1 (0.01)
Rapid review appraisal	1 (0.01)
Guideline assessment	1 (0.01)
Guideline protocol	1 (0.01)
Consensus statement	1 (0.01)
Type of report	
Full manuscript	826 (95)
Conference abstract	45 (5.2)
The most common journals where analyzed articles were published	
*BMJ Open*	31 (3.6)
*Systematic Reviews*	23 (2.6)
*PLoS One*	22 (2.5)
*Medicine*	22 (2.5)
*Journal of Clinical Epidemiology*	19 (2.2)
Chinese Journal of Evidence-Based Medicine	14 (1.6)
Cochrane Database of Systematic Reviews	13 (1.5)
Evidence-based Complementary and Alternative Medicine	11 (1.3)
International Journal of Environmental Research and Public Health	11 (1.3)
AMSTAR version used	
AMSTAR 2	451 (52)
AMSTAR	382 (44)
R-AMSTAR	31 (3.6)
Both AMSTAR and AMSTAR 2	5 (0.006)
Both AMSTAR and R-AMSTAR	1 (0.001)
Unclear whether AMSTAR or AMSTAR 2 was used	1 (0.001)
References used to support the use of AMSTAR more than two times (*N* = 382)	
Shea et al., 2007, BMC Medical Research Methodology [1]	216 (57)
Shea et al., 2009 [5]	94 (25)
Shea et al., 2007, PLoS One [6]	20 (5.2)
Shea et al., 2017 [2]	11 (2.9)
AMSTAR web site	6 (1.6)
Pieper et al., 2015 [7]	6 (1.6)
Sharif et al., 2013 [8]	6 (1.6)
Pollock et al., 2017 [9]	4 (1.0)
Xiong et al., 2009 [10]	3 (0.8)
References used to support the use of AMSTAR 2 (*N* = 451)	
Shea et al., 2017 [2]	396 (88)
Shea et al., 2007, BMC Medical Research Methodology [1]	14 (3.1)
Shea et al., 2009 [5]	9 (2.0)
AMSTAR web site	8 (1.8)
Zhang et al., 2018	3 (0.07)
Lorenz et al., 2019 [11]	2 (0.04)
Shea et al., 2007 PLoS One [6]	2 (0.04)
Ge et al., 2017 [12]	2 (0.04)
Banzi et al. 2018	1 (0.02)
Biondi-Zoccai, 2016 [13]	1 (0.02)
Brouwers et al., 2010 [14]	1 (0.02)
Ciapponi, 2017 [15]	1 (0.02)
Pieper et al., 2014 [16]	1 (0.02)
Pollock et al., 2017 [9]	1 (0.02)
Tian et al., 2017 [17]	1 (0.02)
Xiong et al., 2009 [18]	1 (0.02)
Yan et al., 2018 [19]	1 (0.02)
References used to support the use of R-AMSTAR (*N* = 12)	
Kung et al., 2010 [20]	23 (75)
Shea et al., 2017 [2]	3 (9.7)
Shea et al., 2007, BMC Medical Research Methodology [1]	3 (9.7)
Dosenovic et al., 2018 [21]	
Rotta et al., 2015 [22]	1
Shea et al., 2007 PLoS One [6]	

^a^The percentages for each variable may not add up to 100 due to rounding

AMSTAR 2, the new version of the tool, was used by 52% of the studies, while AMSTAR, the old version of the tool, was used by 44% of the studies (Table 1). In 2018, 81% of the analyzed studies used AMSTAR, while 16% used AMSTAR 2. In 2019, 52% used AMSTAR, while 44% used AMSTAR 2. Among articles published in 2020, 28% used AMSTAR, while AMSTAR 2 was used by 69%.

There were 31 studies that used Revised AMSTAR (R-AMSTAR) proposed by Kung et al. in 2010 [20]. Six studies used two of these tools – five studies used both AMSTAR and AMSTAR 2, while one study used AMSTAR and R-AMSTAR (Table 1).

In the studies that used two tools, we analyzed whether the authors provided an explanation. Sharma et al. [23] justified this by reporting that just before manuscript submission AMSTAR 2 was published, so they added analysis of AMSTAR 2 too. McGuire et al. [24] did not explicitly explain why they used both tools. In the methods, they described features of the tools, and in the results, they mentioned that certain analyses were not conducted with AMSTAR 2 [quote] “*as this instrument is not a numerical scoring system*.” [24]. Kim et al. compared assessments with AMSTAR and AMSTAR 2 with the following explanation [quote]: “*it is not clear whether similar methodological quality evaluations can be performed for the same research because AMSTAR and AMSTAR 2 have a different amount of evaluation items, different item contents, evaluation methods, and evaluation results calculation methods*” [25].

De Santis et al. compared scores on AMSTAR and AMSTAR 2 with the following justification [quote]: “*Since AMSTAR and AMSTAR2 differ substantially, it remains unclear if they produce similar quality ratings for systematic reviews in healthcare*” [26]. Jeyaraman et al. used both AMSTAR and AMSTAR 2 without any comments or explanations for using both tools [27].

A study [28] that used both AMSTAR and R-AMSTAR explained it as follows [quote] “*We also used the revised version of AMSTAR (R-AMSTAR), which assigns an overall quality score to the systematic review”* [28].

### Information sources referenced to support the use of different tools

The studies used as many as 41 different information sources as references to support using AMSTAR, R-AMSTAR, or AMSTAR 2. The median number of references used for these tools was 1 (range: 0 to 3).

Studies that used only the original AMSTAR (*N* = 382) reported 31 different references to support the use of the tool. The majority (*N* = 216; 57%) referenced the 2007 study in which the tool was first described by Shea et al. (Table 1). Other most commonly used references to support the use of the original AMSTAR tool included references by Shea et al. from 2007 and 2009 describing further testing of the tool, or a reference to the AMSTAR website. Multiple authors cited studies of other authors examining AMSTAR. Eleven studies used the original AMSTAR but erroneously referenced Shea et al. manuscript from 2017 that described AMSTAR 2 (Table 1).

Authors of studies that used only AMSTAR 2 (*N* = 451) used 17 different references to support its use. The majority (88%) referenced the article of Shea et al. from 2017, in which the tool was described (Table 1). However, multiple authors also used references to the article describing the original version of the tool [1] or other studies that were published before 2017. One study referenced the article about AGREE II tool [14] instead (Table 1).

Among 31 studies that used only R-AMSTAR, 23 referenced the study of Kung et al. from 2010, in which R-AMSTAR was described [20], while others used references to AMSTAR or AMSTAR 2, or even references to other works that have used R-AMSTAR (Table 1).

### Survey results

There were 354 manuscripts eligible for an author survey. In 11 articles, e-mail address of a corresponding author was not reported. Thus, we invited 343 authors to participate in the survey. Nineteen e-mails returned undelivered. Of the remaining 324 authors, 88 responded (27% response rate). Among responders, 79 responded via Google Forms, and 9 via e-mail. The respondents had a median 13 years of research experience in the field (range: 1 to 25 years).

Among the 88 participants, 68 (77%) indicated that they were aware that AMSTAR 2 was published. Ten (11%) authors in the sample indicated that editors or peer-reviewers asked them to do AMSTAR 2.

Among the 68 participants who were aware of AMSTAR 2, 41 (60%) indicated that they had heard that AMSTAR 2 was published before submitting their manuscript to a journal. Twenty-five (38%) of those 68 participants indicated that they considered using AMSTAR 2 in their manuscript instead of AMSTAR. Reasons for not using the AMSTAR 2 were provided by 44 authors, as shown in Table 2. The authors indicated that they did not use AMSTAR 2 because its psychometric properties were not established at the time, their protocol was already established, their data collection/analysis was completed before AMSTAR 2 was published, they were not aware of AMSTAR 2, did not have time to do another analysis, it was lengthier than the original AMSTAR, editors and peer-reviewers did not request it (Table 2).

**Table 2:** Authors’ reasons for not using AMSTAR 2

Reason	N
We were not aware that AMSTAR 2 was published	9
We have finished the quality assessment with AMSTAR already	7
AMSTAR 2 was not yet published at the time we developed our protocol and submitted our manuscript for peer review	4
Lengthier than AMSTAR 1	4
Familiarity with the previous tool and based on the pre-study consensus	3
Psychometric properties of AMSTAR 2 were not established at that time	2
AMSTAR 2 came out after we completed data collection using AMSTAR	2
I used R-AMSTAR in my study	2
We had already developed the protocol and started the study	1
We started our study long before the AMSTAR 2 was published, and editors or peer-reviewers did not request that we use AMSTAR 2.	1
AMSTAR was still used by other Authors	1
Because our protocol in which we decided on AMSTAR was published prior to the release of AMSTAR 2. Moreover, we had reason to believe that the raking of the included studies would not change substantially by the ude of AMSTAR 2	1
Because the search was performed till March 2017 and critical appraisal afterwards, which corresponded to have the final manuscript done before AMSTAR 2 getting published	1
I did not hear about it on time	1
I heard about AMSTAR 2 after the paper was submitted	1
It was published just as I was submitting my article and so did not make decision to change	1
The article I published was part of my doctoral thesis, which had a stipulated time for homologation of the defense, which prevented me from making changes to use AMSTAR 2. Really, time prevented me from using the instrument.	1
Too close to submission (no time to do the analysis again)	1
We had finalized our review when we knew about AMSTAR 2. It was a systematic overview of systematic reviews of observational studies (not RCTs). We did not feel that the AMSTAR 2 was adding to our assessment.	1
We started the study in 2016. By the time AMSTAR 2 published in 2017, we completed data extraction and analysis and decided to proceed	1
Not in the published study, but have used AMSTAR 2 in a subsequent study.	1
Amstar 2 was judged as less useful than amstar 1 for our QA because several of the new questions were not relevant to our data. It seemed to be more tailored for a specific type of meta-analysis or review.	1
One of the papers we included in the rapid review was a review of systematic reviews, which had employed AMSTAR I for assessing SR. That is the reason we decided to use this instrument instead AMSTAR II.	1
AMSTAR 2 does not allow classification into high, medium or low quality.	1

When asked are there any barriers to the uptake of the AMSTAR 2 that they have experienced or that someone else might experience, 68 participants responded. However, the majority indicated that there were no barriers. Those who identified barriers mentioned the following: lack of quantitative aspect of scoring with AMSTAR 2, lack of awareness about the tool, length (more time needed to use it), lack of familiarity with the tool, difficulties with a specific item (Table 3).

**Table 3:** Barriers towards use of AMSTAR 2

Barrier	N
AMSTAR 2 has difficulty distinguishing the quality between systematic reviews so it is not good for qualitative research. Assessment using AMSTAR 2 does not provide a quantified final information that can be directly compared between studies.	7
It is longer, so that may be the matter of using more time to do it compared to AMSTAR	6
Lack of awareness	4
Lack of familiarity with the tool and uncertainty about how it is different or better than AMSTAR	3
The quality of systematic reviews evaluated using the AMSTAR 2 tool is almost always of low or very quality	2
It is new, so people need to invest time to learn how to do it, while they are probably already familiar with the old version of AMSTAR	1
I have difficulty with the item on publication bias, when it was not possible to carry out this analysis due to the small number of studies. There is no suitable option.	1
AMSTAR 2 is not widely used yet	1
Better operationalization of variables (more detailed) to ensure better inter-rater reliability	1
Confusing instructions, unnecessarily complex or specific questions, and no weights for questions even though certain recommendations are more important than others.	1
It is not clear from the AMSTAR 2 paper or guidance what should be done with a score of ‘Partial yes’	1
AMSTAR 2 suggests that searching grey literature is optional (‘sometimes important’) - this seems to be in opposition to the Cochrane Handbook.	1

## Discussion

In this study, we found that in articles published in 2018-2020, just over half of the authors used AMSTAR 2 (52%). As many as 44% of the articls still used old version of the AMSTAR tool despite the publication of AMSTAR 2 in September 2017. AMSTAR was used in more than half of articles published in 2018 and 2019, but its use declined to 28% in year 2020. Few authors used R-AMSTAR, and some even combined use of two of these instruments.

New tools in the field of methodological studies are continuously developed and updated. For AMSTAR 2, it has already been reported that this is a better version of the tool. However, the authors of OSRs and methodological studies appraising the quality of SRs may consider that AMSTAR 2 requires more work since it has more items (11 items in AMSTAR, compared to 16 items in AMSTAR 2) and requires authors to study instructions and background information for the new tool.

We are aware that it takes time for the preparation and publication of manuscripts and that our analysis started with manuscripts published less than 4 months after the publication of AMSTAR 2. However, even if manuscripts were in the final stages of preparation and peer-review, authors, editors and peer-reviewers could have decided that AMSTAR 2 should be used instead. In our sample, 11% of authors indicated that editors or peer-reviewers asked them to use AMSTAR 2.

One of the studies that used both AMSTAR and AMSTAR 2 reported that the AMSTAR 2 was added to the analysis because it was published just before their manuscript submission [23]. Thus, the period we analyzed could be considered an analysis of AMSTAR 2 uptake in the early period after its publication. It is anticipated that the use of the first version of AMSTAR will decline further in the coming years.

The majority of analyzed studies used references to articles about the development of analyzed tools to support their use. We observed minor discrepancies and errors in that respect; some authors used references to a different tool than the one they used. Some authors did not use references to articles describing the development of AMSTAR, AMSTAR 2, and R-AMSTAR; instead, they used references of other author groups, in which the tool was further tested or simply used on another sample of studies.

It needs to be emphasized that R-AMSTAR is different from AMSTAR and AMSTAR 2. While AMSTAR and AMSTAR 2 were developed by the same research group, R-AMSTAR was developed by another research team [20]. Kung et al. created the R-AMSTAR because AMSTAR did not include quantifiable assessments of systematic review quality. Thus, R-AMSTAR aimed to “quantify the quality” of SRs [20]. The R-AMSTAR used 11 original domains of AMSTAR, but each domain is scored with 1 to 4 points. Thus, the potential overall score on the tool may range from 11 (minimum) to 44 (maximum). An SR with a total score of 11 did not satisfy any of the AMSTAR criteria, while a score of 44 denotes an SR that satisfied all the methodological criteria of AMSTAR, in every domain [20]. The validity of R-AMSTAR has been questioned because it is difficult to weigh the individual items in terms of relative importance while calculating the final score. Thus, it has been suggested that the measurement properties of the R-AMSTAR should be studied further [7].

Our author survey indicated that multiple authors were aware of AMSTAR 2 but did not use it in their study because they already established their protocol, and their data collection was well underway. Few were asked by editors and peer-reviewers to use the new version of the tool. Even those who were not aware of the new tool could have used it if the editors and peer-reviewers asked them to do so.

Few authors identified barriers towards the use of AMSTAR 2. Thus, it is anticipated that the authors will continue the trend of abandoning the initial version of the AMSTAR and using the AMSTAR 2 in the future years.

Based on the findings of our study, it is worth considering what could be done to increase the use of new versions of the methodological tools when they become available. Namely, journals could use their instructions for authors to indicate that they expect authors to use the new versions of the tool. Furthermore, editors and peer-reviewers could request authors to use the new versions of the tool. Finally, educators in research methodology and evidence synthesis should include novel critical appraisal tools into their curricula.

Another point for improvement is the correct referencing of the tools. This study showed that many authors use erroneous references to support the use of the chosen tool. For example, multiple authors who used AMSTAR provided references to AMSTAR 2 and vice versa. Furthermore, some authors did not reference the articles describing the development of these tools; instead, they cited other studies that have used the tool, which is not optimal. This issue of correct referencing would benefit from more attention from authors, peer-reviewers and editors.

Additionally, our study points to the important issue of difficulties in contacting corresponding authors. We could not contact 8% of the tentative participants either because the e-mail address was not available in the published manuscript or because the message returned undelivered from the recipient’s e-mail address. Contact via e-mail is now considered the norm. Thus, the lack of contact e-mails in published manuscripts and e-mail decay is worrying because it means that these authors cannot be easily contacted for research-related purposes. Already in 2006, it was observed that one in four e-mail addresses becomes invalid, seriously impacting the ability of researchers to communicate and exchange material [29].

The research community is continuously analyzing AMSTAR and its new version [21, 30, 31], and our study is another contribution in that direction. Furthermore, since the tools are usually proposed by a group of authors and then published, we consider it beneficial that the research community questions and monitors the proposed methodological tools. Methodological research can ultimately help advance medical research [32].

A limitation of our study includes the use of Google Forms to conduct the survey. The advantage of Google Forms is that they are free to use, unlike proprietary software for surveys. However, several invited authors from China alerted us that they could not use Google. Thus, we combined an e-mail survey with a Google Form survey. Some authors could be deterred from providing answers via e-mail because of a loss of anonymity. Based on our experience, authors targeting international audiences for their surveys should check whether their survey platform may have geographical obstacles.

Another limitation is a modest response rate in our survey; 27% of the authors with delivered e-mails responded to the survey invitation. This response rate can be considered adequate for an unsolicited online survey received from an unfamiliar researcher.

Furthermore, in this study, we analyzed the frequency of the use of AMSTAR tools, ad we did not address methodological issues such as the advantages or disadvantages of the AMSTAR 2 compared to AMSTAR. We did not survey authors that had used AMSTAR 2 to ask them why they used the new tool, and whether they found AMSTAR 2 better than AMSTAR in terms of comprehensiveness of the evaluation, clarity of the domains, presence of guidance for use, or their perceptions about the limitations of the new tool. This could be a topic for further research. New studies can also explore the period after 2018-2020 to assess further adoption of AMSTAR 2.

In conclusion, in articles published in 2018-2020 that were submitted to a journal after AMSTAR 2 tool was published, almost half of the authors (44%) still used AMSTAR, the old version of the tool. However, the use of AMSTAR has been declining in each subsequent year. Few barriers towards using AMSTAR 2 were identified, and thus it is anticipated that the use of AMSTAR will continue to decline.

## Supplementary Information

**Additional file 1.**

**Additional file 2.**

**Additional file 3.**

## Data Availability

All raw data collected within the study are reported in Supplementary file 3.
