# Supplementary material for: Adopting AMSTAR 2 critical appraisal tool for systematic reviews: speed of the tool uptake and barriers for its adoption
Source: BMC Med Res Methodol. 2022 Apr 10;22:104. doi: 10.1186/s12874-022-01592-y (PMC8996416; doi:10.1186/s12874-022-01592-y)
Supplement: Supplementary file 1 — Additional file 1. [file 12874_2022_1592_MOESM1_ESM.docx]

**Supplementary file 1. Text of the e-mail sent to potential study participants**

Title of the message:

Your opinion about the uptake of AMSTAR 2 (it will take only few minutes of your time)

Text of the message:

Dear *Participant* [*insert title and name here*],

We are researchers from Cochrane Croatia, and we are conducting a study about the adoption of AMSTAR 2, and barriers related to AMSTAR 2 adoption. We are contacting you because you have recently published a study in which you used AMSTAR:

*[Name of the study]*

We have several questions for you, which may require **only** 1-3 minutes of your time to answer. Your responses will be anonymized and analyzed in aggregate form. We would be very grateful for few minutes of your time and your responses to these questions.

Link to the survey:

<https://docs.google.com/forms/d/e/1FAIpQLSezWQ08c0A9ccpCgKMlQwmFZwfqFN689puNrZOM9i5saq95sw/viewform>

More detailed information about this study can be found at the bottom of this message.

Thank you very much for your consideration.

Sincere regards,

Name and surname

Additional information about this study:

Principal investigator: Prof. Livia Puljak, MD, PhD

Contact of the principal investigator: [livia.puljak@unicath.hr](mailto:livia.puljak@unicath.hr)

Ethics approval: The study was approved by the Ethics Committee of the Catholic University of Croatia.

Informed consent: Based on the protocol approved by the Ethics Committee, entering the survey and responding to the study questions will be considered as a consent to participate.

Intention to publish: Our research team intends to publish anonymized data collected in this study. Your name as a study participant will never be mentioned in any publicly available information about this study.

If you have any questions about this study, please do not hesitate to contact us.
